# Interference With VIP to Distinguish Between Real and False VIPoma: National Study From the French Endocrine Tumors Group

**DOI:** 10.1210/jendso/bvae102

**Published:** 2024-05-23

**Authors:** Benjamin Chevalier, Delphine Bonnet, Come Lepage, Marine Perrier, Françoise Borson-Chazot, Juliette Abeillon, Jean Bernard Delobel, Arnaud Jannin, Julien Hadoux, Magalie Haissaguere, Catherine Lombard-Bohas, Thomas Walter, Laurence Chardon

**Affiliations:** Department of Nuclear Medicine, Lille University Hospital, Lille 59000, France; School of Medicine, University of Lille, Lille 59000, France; Department of Internal Medicine and Digestive Diseases, CHU Toulouse, Toulouse 31059, France; Gastroenterology and Digestive Oncology, Hôpital Universitaire Le Bocage, Dijon 21079, France; Université Reims Champagne-Ardenne, Department of Gastroenterology and Digestive Oncology, Reims University Hospital, 51092 Reims, France; Hospices Civils de Lyon, Hôpital Louis Pradel, Fédération d’Endocrinologie, Lyon 69500, France; Hospices Civils de Lyon, Hôpital Louis Pradel, Fédération d’Endocrinologie, Lyon 69500, France; Hôpital Yves le Foll, Service de Gastroentérologie, Saint Brieuc 22000, France; School of Medicine, University of Lille, Lille 59000, France; Department of Endocrinology, Diabetology and Metabolism, Lille University Hospital, Lille 59000, France; CANTHER—Cancer—Heterogeneity Plasticity and Resistance to Therapies, University of Lille, UMR9020-U1277—CNRS, INSERM, CHU Lille, Lille 59000, France; Department of Nuclear Medicine and Endocrine Oncology, Gustave Roussy Cancer Center, Villejuif 94800, France; Endocrinology and Endocrine Oncology Department, Haut Leveque Hospital, University Hospital of Bordeaux, Bordeaux 33600, France; Hospices Civils de Lyon, Hôpital Edouard Herriot, Service d’Oncologie, Lyon 69003, France; Hospices Civils de Lyon, Hôpital Edouard Herriot, Service d’Oncologie, Lyon 69003, France; Service de Biochimie, Groupement Hospitalier Est, Hospices Civils de Lyon, Bron 69500, France

**Keywords:** vasoactive intestinal peptide, neuroendocrine tumor, analytic interference

## Abstract

**Background:**

Vasoactive intestinal peptide (VIP)-secreting tumors (VIPomas) are digestive neuroendocrine tumors in which the hormonal secretion is life-threatening. Biological confirmation is obtained by demonstrating an elevation in plasma VIP, usually using radioimmunoassay (RIA). In some cases, analytical interference is suspected. We developed 3 different techniques to detect interference in VIP RIA.

**Methods:**

Three techniques were used: RIA after Sephadex column chromatography separation, RIA after polyethylene glycol precipitation, and ^125^I-labeled VIP binding test. We included patients with suspicion of false positive VIP (FPV) elevation. We then compared results with those of a group of “real,” proven VIPoma (RV).

**Results:**

A total of 15 patients with FPV elevation and 9 RV patients were included. Interference was detected in all FPV patients vs none in RV. Clinical and biochemical parameters did not differ between FPV and RV patients, but VIP concentration in RIA was significantly higher in FPV patients than in RV patients (228 pmol/L vs 66 pmol/L, *P* = .038). Using a ^125^I-labeled VIP binding test, median proportion of radioactivity in the pellet was significantly higher in FPV than in RV patients (53% vs 13%, *P* < .0001). A 20.5% threshold presented excellent performances (sensitivity 100% [79.6-100], specificity 100% [70.1-100]).

**Conclusion:**

We developed 3 different laboratory techniques to reveal interference in RIA VIP assays. The diagnostic performance of all 3 was excellent. These techniques must be employed in cases of discordance between VIP elevation and clinical presentation.

Vasoactive intestinal peptide (VIP) is a 28–amino acid monomeric peptide (molecular weight of 3 kilodalton), mainly produced by neurons in the central and peripheral nervous system, notably in the nerve terminals of the gastrointestinal tract [1]. It mediates multiple physiological actions, such as vasodilating action, epithelial secretion, absorption in the gastrointestinal tract, and muscular contraction modulation. It may also play a role in the pathophysiology of several inflammatory or metabolic diseases [2].

VIP-secreting tumors (VIPomas) are well-differentiated functional digestive neuroendocrine tumors (NETs), located in the pancreas in 80% of instances [3]. They are particularly rare with an estimated incidence of 0.1 to 0.2 cases per million per year. The clinical phenotype, which could be life-threatening, typically includes profuse aqueous diarrhea, hypokalemia, and achlorhydria [4-7]. VIPomas classically present as bulky lesions with a median diameter of about 5 cm and metastatic extension in 60% to 75% of cases, according to different series [7, 8]. Biological confirmation is obtained by demonstrating an elevated plasma VIP, the most commonly used assay method being radioimmunoassay (RIA) [9].

VIP may be systematically screened during exploration of potential pancreatic NET or in asymptomatic patients with a genetic predisposition to develop endocrine tumors, such as type 1 multiple endocrine neoplasia (MEN1) [10]. As in any assay, some results can be intriguing or questionable because they are not in accordance with clinical features or other biological results, or with previous results, all of which could lead to the suspicion of assay interference [11, 12]. Notably in RIA, the main interference is due to antibodies that bind to samples and/or reagents, like heterophilic antibodies or antibodies that lead to the formation of macro-complexes, resulting in false positive or false negative results. In these situations, it is therefore necessary to develop tools to identify this interference, to avoid the deleterious consequences that an erroneous diagnosis of VIPoma would represent for the patient.

In the present study, we developed 3 different techniques to reveal interference in standard VIP RIA. These techniques were applied in patients with elevated plasma VIP levels, according to RIA assay in which there was a suspicion of interference. Finally, we compared baseline characteristics of these patients with those of patients with pathologically proven VIPomas to identify predictive factors of interference.

## Methods

### Patient Selection

This retrospective study was conducted according to the principles of the Declaration of Helsinki. Patients with suspected false positive VIP elevation (FPV), belonging to the national French ENDOCAN-RENATEN network, were included. Suspicion of false positive VIP elevation was defined as (i) a pathological elevation of VIP without VIP-associated clinical signs/symptoms; and (ii) a discordance between the level of VIP elevation and VIP-associated clinical signs/symptoms. Moreover, the Lyon Digestive Oncology Department database was screened, approved by CNIL (Commission Nationale de l'Informatique et des Libertés) and its ethical committee, on the November 6, 2015 (n°15-111), to compare characteristics of FPV patients with those of patients with real, histologically proven, VIP-secreting NET (RV). Written information was given to each patient included in the study. Their consent is not required by French law, but patients are informed about their right to withdraw their data from the cohort.

The following data were collected: age, sex, presence of a genetic predisposition to develop NETs, clinical presentation, and biochemical, hormonal, and morphological explorations. Severity of diarrhea was graded according to Common Terminology Criteria for Adverse Events (CTCAE, Version 5.0) [13]. A strong probability of VIPoma was defined as the presence of significant (≥ grade 2) diarrhea +/− hypokalemia, VIP elevation with conventional immunological assay and the presence of a macroscopic NET at computed tomography (CT)/magnetic resonance imaging/nuclear imaging.

Regarding RV patients, extension at diagnosis and pathological features were also collected. These patients were managed according to the standard of care [14-16].

### VIP Assay Methods

Reference VIP assay and methods to detect interference were developed using plasma of patients (FPV and RV) managed in centers specialized in NET management, between 2017 and 2021.

Blood was collected in tubes containing EDTA and aprotinin, and samples were centrifuged at +4 °C to separate plasma, which was then frozen within 1 hour before being stored at −20 °C until assayed.

### Reference VIP Assay

The main VIP assay carried out in France is a competitive RIA in which VIP to be measured in samples competes with ^125^I-labeled VIP to bind to a rabbit antiserum raised against VIP-albumin conjugate (VIP RIA, Diasource Immunoassays® S.A., Belgium, Diasource Cat# RB311, RRID:AB_3095296). Laboratory inter-assay variation of the RIA is 10.6% at 15 pmol/L and 6.8% at 52 pmol/L, and the intra-assay variation is 8.6% and 5.2% respectively. The assays were conducted in the biochemistry department (Groupe Hospitalier Est of the Hospices civils de Lyon, Bron, France), according to the manufacturer's instructions.

### Methods to Test Interference in the VIP RIA

#### RIA after Sephadex column chromatography separation

**Figure 1.**
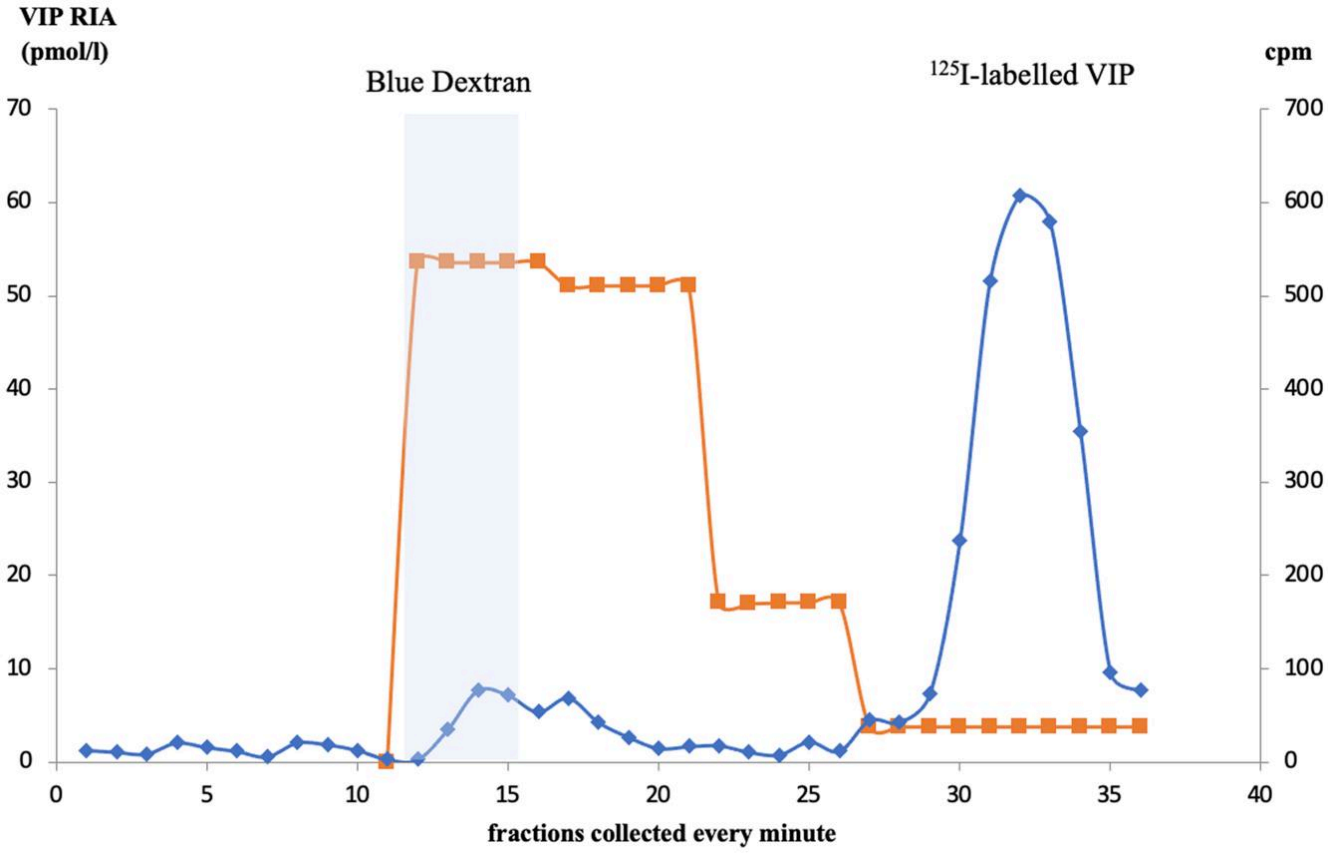
Sephadex G50 column chromatography to detect false VIP interference on RIA. (flow rate of 1 mL/min). Chromatography of a false positive VIP plasma (Cpm: counts per minute): VIP RIA of each fraction collected reflects VIP antigenicity in fractions of high molecular weight. Blue line represents ^125^I-labeled VIP; orange plot the blue dextran.

In accordance with Koch et al [9], 0.5 mL of sample was applied to a Sephadex G50 column (Sigma-Aldrich Merck) equilibrated with a phosphate-buffered saline containing 0.5% bovine serum albumin (BSA), in order to segregate components according to their molecular weights (see Fig. 1). Calibration was performed with blue dextran and ^125^I-labeled VIP. Chromatography is conducted at a constant flow rate of 1 mL/min and fractions were collected every minute for subsequent VIP RIA assays.

#### RIA after polyethylene glycol precipitation

VIP concentrations were determined using the RIA from the supernatant obtained after polyethylene glycol (PEG) precipitation of antigen/antibody complexes, VIP/anti-VIP. In detail, 100 µL of plasma was incubated with 100 µL of BSA for 24 hours. Samples were incubated using 300 µL of PEG 25% for 10 minutes. Antigen/antibody complexes are then separated after centrifugation at 3700*g* for 15 minutes at 4 °C, and the supernatant removed for analysis.

#### ^125^I-labeled VIP binding test

The intrinsic binding capacity of samples, because of the presence of anti-VIP antibodies, was evaluated by incubating 100 µL of plasma with 100 µL of ^125^I-labeled VIP over 18 hours at +4 °C, then precipitated after a 10-minute incubation using 300 µL of PEG 25%. After mixing and centrifugation at 3700*g* for 15 minutes at 4 °C, the radioactivity of ^125^I contained in the pellet was measured with a gamma-counter (Wizard 2470 Perkin Elmer) and compared with the radioactivity of the whole sample.

#### Controls

Plasma controls were consistently tested and were prepared as follows: the negative control was selected from patient without any pancreatic neoplasm, who presented a normal result for the VIP; the positive control was prepared by adding antiserum anti-VIP to a negative plasma.

## Statistical Analysis

Qualitative variables are presented as count (percentage), and quantitative variables are expressed as median [range]. For qualitative variables, groups were compared using the Fisher exact test due to the small sample size and for quantitative variables using the Mann-Whitney-Wilcoxon test. Receiver operator characteristic (ROC) analysis and 95% CI were used to identify the optimum threshold that could distinguish FPV and RV regarding VIP concentration on immunoassay and the VIP binding rate. A *P* value of < .05 was considered statistically significant. GraphPad Prism, version 8.2.1 (GraphPad Software) was used for the statistical analyses.

## Results

### Initial Explorations and Development of Techniques to Detect Interference in VIP RIA

The plasma of 3 patients with suspected FPV (patients 1-3, all asymptomatic) and plasma of 1 documented RV were first analyzed using the 3 different techniques to test the hypothesis of assay interference on immunoassays (Table 1). The VIP level on RIA was > 120 pmol/L in all 3 patients; after dilution the VIP level was of 2850 pmol/L in patient 1 (1/51 dilution), 1252 pmol/L in patient 2 (1/100 dilution), 468 pmol/L in patient 3 (1/41 dilution), and 109 pmol/L in the RV sample.

**Table 1.** Exploration of VIP interference with 3 different techniques in the first 3 patients with suspected of false positive VIP elevation and in a patient with proven VIPoma

	FPV			RV
	Patient 1	Patient 2	Patient 3	Patient 1
Clinical context	ø pancreatic tumor	ø pancreatic tumor	Pancreatic tumor on CT scan,	Metastatic pancreatic tumor
			MEN1 syndrome	
	Asymptomatic	Asymptomatic	Asymptomatic	Symptoms related to VIP secretion
VIP dosage	> 120 pmol/L (pure plasma)	> 120 pmol/L (pure plasma)	> 120 pmol/L (pure plasma)	109 pmol/L
(RIA)	> 1320 pmol/L (1:11 dilution)	724 pmol/L (1:10 dilution)	439 pmol/L (1:11 dilution)	
	2850 pmol/L (1:51 dilution)	2977 pmol/L (1:50 dilution)	468 pmol/L (1:41 dilution)	
		1252 pmol/L (1:100 dilution)		
RIA after Sephadex	Detection and dosage of VIP	Detection and dosage of VIP	Non conclusive	Detection and dosage of VIP
column chromatography separation	High molecular weight (10-20 kDa)	High and medium molecular weight		Low molecular weight (4-5 kDa)
RIA after polyethylene glycol (PEG) precipitation	100 pmol/L (∼3%)	< 3.8 pmol/L	< 3.8 pmol/L	130 pmol/L
^125^I-labeled VIP binding test	67%	87%	88%	6%
Interpretation	3/3 positive tests	3/3 positive tests	2/3 positive tests	0/3 positive test
	Presence of anti-VIP antibodies	Presence of anti-VIP antibodies	Presence of anti-VIP antibodies	Absence of anti-VIP antibodies

Abbreviations: FPV, false positive VIP elevation; RIA, radioimmunoassay; RV, real, histologically proven VIPoma; VIP, vasoactive intestinal peptide; VIPoma, VIP-secreting tumor.

After Sephadex column chromatography, VIP level on RIA, carried out on the different fractions collected from the 3 suspected FPV patients, revealed VIP antigenicity in fractions of high molecular weight (10 to 20 kDa). Moreover, in the plasma of the FPV suspected patients 1 and 2, the concentration of the fractions corresponding to ^125^I VIP was low, reflecting the presence of antigen-antibody complexes including VIP. The result in patient 3 was nonconclusive. VIP was observed in low molecular weight fractions (4-5 kDa) in the plasma of the RV patient, but not in the high molecular weight fractions.

After the removal of antigen-antibody complexes through PEG precipitation, the VIP level on RIA dropped dramatically in the plasma of patient 1 (100 pmol/L, equivalent to ∼3% of the value with conventional RIA) and normalized in the plasma of patient 2 and 3 plasma (< 3.8 pmol/L), but not in the positive control and in RV patient (130 pmol/L).

The ^125^I-labeled VIP binding test showed an intrinsic binding capacity against ^125^I-VIP; in patient 1, 67% of the total radioactivity was observed in the pellet, 87% in patient 2, and 88% in patient 3. In contrast, 6% of the total radioactivity was observed in the pellet of the RV patient. Therefore, the coherent results found using these different techniques leads to the conclusion that anti-VIP antibody-related interference was present (Table 1).

Because chromatography is both time-consuming and expensive, it was decided to use only the ^125^I-labeled VIP binding test on all the patients.

### Characteristics of All Patients With Suspicion of False Positive VIP Elevation

Including the 3 first patients mentioned above, a total of 15 patients were included. Three other patients had suspected FPV and were screened for VIP interference but were not included in the present study because of the absence of clinical data. Of the included patients, 11 (73%) were female and the median age was 61 years (19-83). The VIP assay was carried out as a regular hormonal follow-up: in 2 patients with MEN1 syndrome, in 1 patient during the exploration of a cutaneous lesion, in 6 patients during the exploration of diarrhea, and in 3 patients for a suspicion of VIPoma. Most patients 12 (80%) were symptomatic; the most reported symptoms were diarrhea in 9 (60%) patients, fatigue in 6 (40%), and weight loss in 6 (40%). Diarrhea grading ≥ G2 (ie, an increase of at least 4-6 stools per day) was documented in 8/9 FPV patients. Median potassium levels were 4.0 mmol/L and 3 (20%) patients presented with hypokalemia (Table 2).

**Table 2.** Baseline clinical and biochemical characteristics of patients with proven VIPoma and of patients with suspected of false positive VIP elevation

	FPV	RV	*P* value
	n = 15	n = 9	
Median age in years (range)	61 (19-83)	49 (30-85)	NS
Female, n (%)	11/15 (73)	3/9 (33)	
Multiple endocrine neoplasia type 1, n (%)	2/15 (13)	0/9 (0)	
Symptoms at diagnosis, n/N (%)	12/15 (80)	8/9 (89)	
Diarrhea	9/15 (60)	6/9 (67)	
Diarrhea grading (% > = G2)	8/9 (89)	5/6 (83)	
Median diarrhea grading	2	2	
Abdominal pain	5/15 (33)	7/9 (78)	
Nausea or vomiting	2/15 (13)	0/9 (0)	
Fatigue	6/15 (40)	4/8 (50)	
Weight loss	6/15 (40)	4/8 (50)	
Biological laboratory			
Median potassium levels [mmol/L] (range)	4 (2.8-4.5)	3.6 (2.1-4.1)	
Hypokalemia n/N (%)	3/15 (20)	3/7 (43)	
Median bicarbonates levels [mmol/L] (range),	25.5 (11-35)	25 (21-28)	
Median glomerular filtration rate [mL/min/1.73 m^2^]	91 (48-114)	91 (82-152)	

Abbreviations: FPV, false positive VIP elevation; RV, real, histologically proven VIPoma; VIP, vasoactive intestinal peptide; VIPoma, VIP-secreting tumor.

The median VIP levels was 228 pmol/L [range, 52-15 000] (Table 3), and 3 (20%) patients presented a concomitant elevation of chromogranin A. No patient presented suspicion of digestive hormonal co-secretion. Pancreatic NET was already diagnosed in 3 patients. The evolution of symptoms was known for 5 patients: 2 were being treated with somatostatin analogs, without symptom modification. One patient received exogenous pancreatic enzymes, while another had proton pump inhibitor. In both cases this led to an improvement of the symptoms. Finally, the fifth patient presented spontaneous clinical improvement.

**Table 3.** Baseline endocrine and morphological characteristics of patients with proven VIPoma and in patients with suspected of false positive VIP elevation

	FPV	RV	*P* value
	n = 15	n = 9	
Median VIP [pmol/L] (range)	228 (52-15000)	66 (31-400)	*^a^*
Median VIP [pg/mL] (range)	759 (173-50000)	220 (103-1333)	*^a^*
Median Chromogranin A [ng/mL](range)	60 (21-2237)	81 (35-2991)	NS
Abdominal mass at morphological imaging, n (%)	3/11 (27)	9/9 (100)	*^c^*
Abdominal mass at nuclear imaging, n (%)	2/8 (25)	3/4 (75)	NS
Location of the mass within the pancreas, n	3/3 (100)	9/9 (100)	NS
NET stage, n/N (%)			
Stage I-II	2/3 (67)	2/9 (22)	NS
Stage III	0	2/9 (22)	
Stage IV	1/3 (33)	5/9 (55)	
NET grade, n/N (%)			
NET G1	1/2 (50)	4/9 (45)	NS
NET G2	1/2 (50)	5/9 (55)	
NET G3	0	0	
Median Ki67 in % (range)	5.75 (1.5-10)	6 (2-10)	NS
Size (mm)	25 (21-50)	40 (10-90)	NS
(Diarrhea > = G2 and/or hypokalemia) +	1/15 (6)	6/9 (67)	*^b^*
pancreatic abnormality on CT scan			

Abbreviations: FPV, false positive VIP elevation; G, grade; NET, neuroendocrine tumors; RV, real, histologically proven VIPoma; VIP, vasoactive intestinal peptide.

^*a*^ *P* < .05.

^*b*^ *P* = < .01.

^*c*^=*P* = < .001.

### Comparison With “Real” VIPomas

A total of 9 patients were identified as RV out of the 2250 patients in the database. When comparing FPV patients with RV patients, no significant difference was found regarding clinical or biochemical presentation, especially regarding the severity of diarrhea and median potassium levels. (Table 2).

The median VIP concentration, according to conventional RIA, was significantly lower in RV than in FPV (66 pmol/L vs 228 pmol/L, *P* = .038). In order to identify a reliable threshold above which FPV is highly suspect, ROC curve analysis was performed and identified a 420 pmol/L threshold associated with a 100% sensitivity [67.6-100] but a 46.7% specificity [24.8-69.9].

All 9 patients had a grade 1-2 pancreatic NET (median Ki67 of 6%) and 7 (63%) were at metastatic stage. The proportion of patients with a strong a priori probability of VIPoma was significantly lower in FPV than in RV (1/15 [6%] vs 6/9 [67%], *P* = .003), Table 3).

### Pooled Biological Explorations

**Figure 2.**
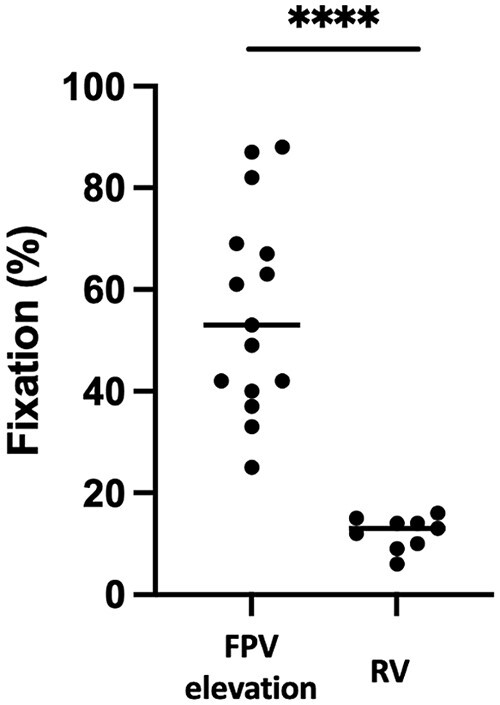
^125^I-labeled VIP binding test. Fraction of radioactivity in the pellet compared to radioactivity in the whole sample; ****=*P* < .0001.

In the 15 patients with suspected FPV, an interference was observed in 100% of samples. Of note, the 3 patients who were not included because of lack of clinical data also presented an interference. No interference was observed in the subsequent 8 RV patients. The median proportion of radioactivity in the pellet was significantly higher in FPV than in RV patients (53% vs 13%, *P* < .0001; Fig. 2). Using the ROC curve, a threshold of 20.5% presented excellent diagnostic performances, with sensitivity of 100% [79.6-100] and specificity of 100% [70.1-100].

Because interference screening was performed in the 2020-2022 period, it represents an estimated mean prevalence of 6 FPV diagnosis a year. In comparison, in our center and during the same period, 370 VIP dosage were performed a year, of which 10 were positive (mean: 3.3 per year).

## Discussion

Using for the first time distinct complementary laboratory techniques, the present study enabled detection of interference in VIP RIA. VIPoma is a life-threatening disease that must be managed in tertiary expert centers. Whenever suspected, multiple tests are carried out and treatment using somatostatin analogs may be implemented.

Over a period of 3 years, we observed 2 times more FPV than real VIPomas. This observation is coherent because of the rarity of these NETs; however, a precise estimation of FPV is quite difficult to make because the expertise of our center, an ENETS Center of Excellence, which is the only center in the country to perform VIP interference screening, therefore likely leads to an overestimation of the prevalence of FPV. To our knowledge, there are no data available in the literature regarding this point; the Liverpool ENETS Center of Excellence published few years ago their experience regarding the frequency and causes of false positive elevated plasma concentrations of fasting gut hormones and found only 2 elevated tests with 1 false positive due to failure to respect fasting state at dosage [17].

We chose to look for interference in atypical clinical situations; the FPV patients included herein did not present the classical clinical-biological feature of VIPoma, principally diarrhea and hypokalemia. Conversely, they showed conflicting features of particularly high VIP concentrations associated to mild signs and symptoms, a situation supposed to be rare, or even nonexistent, because, except for systematic screening, for example in MEN1 patients, VIP dosages are initially performed because of the presence of symptoms, with more or less marked intensity. However, we cannot provide a quantitative estimation.

The interference was even more suspected in patients with no pancreatic mass at imaging, again a situation supposedly rare but also difficult to provide a precise estimation: in our current work, 77% of patients with FPV presented without pancreatic mass vs 0% in real VIPoma. Furthermore, the smallest size of VIPoma observed in our study was 10 mm. In the work of Brugel et al, who provided a national case series of VIPoma and a review of the literature, all patients also presented with pancreatic mass, with a median size of 55 mm and a smallest size of 36 mm [7].

Interestingly, VIP level on RIA in RV patients never exceeded 400 pmol/L in this series, while it could reach up to 15 000 pmol/L in FPV patients, and median values were significantly different. However, due to an overlap between the 2 groups, we were not able to propose a reliable threshold above which FPV should be highly suspected. Furthermore, when clinical or biochemical data were pooled, if each variable is taken independently there was no significant difference between FPV and RV patients. Therefore, it is still important to discuss these cases in multidisciplinary NET Tumor Boards and, in cases of suspected assay interference, to carry out additional blood tests as described in this study.

The 3 methods reported herein confirmed the presence of interference for each plasma sample with suspected false-elevated VIP. In contrast to chromatography, which is time-consuming and expensive, methods based on precipitation with PEG or ^125^I-VIP binding can be easily implemented in any laboratory that has experience in RIA, and we chose to only perform the ^125^I-VIP binding method in our center in cases of suspected of interference in RIA.

This study has some strengths: the assays were centralized in a single center that developed them. These techniques are powerful with excellent sensitivity. Moreover, a comparative control group of patients with proven VIPoma, representative of known literature about the disease, was used, and enabled us to show the high specificity of the tests [5, 6]. In addition, the VIP level on RIA in RV patients herein was consistent with the study and review of literature recently published by Brugel et al [7]. Patients were recruited from numerous referral centers, which limits the inclusion bias and reflects the clinical sense of several different expert physicians. However, the study also had some limitations, principally its retrospective design, which resulted in a lack of precise data in some patients, such as the exact number of stools per day, or the evolution of symptoms in most cases of the false positive VIP elevation. Furthermore, only the ^125^I-VIP binding test, and not the other 2 tests, was used in the majority of patients with FPV. Nevertheless, this does not call into question the results of the research on biological interference in the assay. We cannot ignore a small risk that an occult VIPoma could have been missed—although most VIPomas present as large tumors—and furthermore there was no death in FPV patients that could have been related to a VIP secretion. The last limitation of this study is the small size of this series, which is related to the rarity of the disease.

These techniques are useful tools for clinicians, and should be performed in cases of discordance between VIP elevation in the immunoassay and the clinical presentation, especially if the VIP concentration is above 420 pmol/L. It is essential for clinicians and biologists to discuss and to resolve these cases, because interference can lead to erroneous conclusions and a long list of inappropriate explorations. These unnecessary investigations are stressful for both patients and physicians, and costly for society. In addition, unnecessary treatments may be initiated, with a risk of avoidable side effects (although somatostatin analogs are usually well tolerated).

In conclusion, the present study provides different laboratory techniques with excellent diagnostic performances to reveal interference in RIA VIP assays.

## Abbreviations

FPV: false positive VIP elevation
MEN1: type 1 multiple endocrine neoplasia
NET: neuroendocrine tumor
PEG: Polyethylene Glycol
RIA: radioimmunoassay
ROC: receiver operator characteristic
RV: real, histologically proven VIPoma
VIP: vasoactive intestinal peptide
VIPoma: VIP-producing neuroendocrine tumor
